# Comparisons of the clinical outcomes of Centurion^®^ active fluidics system with a low IOP setting and gravity fluidics system with a normal IOP setting for cataract patients with low corneal endothelial cell density

**DOI:** 10.3389/fmed.2023.1294808

**Published:** 2023-11-23

**Authors:** Yinan Liu, Jing Hong, Xiaoyong Chen

**Affiliations:** ^1^Department of Ophthalmology, Peking University Third Hospital, Beijing, China; ^2^Beijing Key Laboratory of Restoration of Damaged Ocular Nerve, Beijing, China

**Keywords:** Active Sentry, phacoemulsification, corneal endothelial cell density, corneal edema, Alcon Centurion^®^

## Abstract

**Background:**

During cataract phacoemulsification surgery, the Alcon Centurion with Active Sentry can achieve a more stable anterior chamber, which allows a lower intraocular pressure (IOP) setting than the gravity fluidics system. In this randomized controlled trial, we compared these two systems’ damage to the cornea under different IOP settings.

**Methods:**

Seventy-eight eyes of 53 patients with corneal endothelial cell density (ECD) of 500∼1500/mm^2^ were enrolled and randomly divided into the active fluidics system (AFS) group using an Active Sentry handpiece with 30 mmHg IOP setting (40 eyes) and the gravity fluidics system (GFS) group using an Ozil handpiece with 80 cmH_2_O IOP setting (38 eyes). Intraoperative parameters, visual acuity, corneal edema ratio, central corneal thickness (CCT) changes as well as loss rate of ECD were analyzed.

**Results:**

We observed no significant differences in best corrected visual acuity (BCVA), cumulative dissipated energy (CDE), total case time, estimated fluidics usage (EFU) and ophthalmic viscoelastic devices (OVDs) usage between the two groups. The enrolled eyes were further divided into soft nucleus (27 eyes) and hard nucleus (51 eyes) subgroups. And we found less pain complaint during surgeries, lower corneal edema ratio at 1-day and 1-week visit, smaller CCT changes at 1-day visit and lower ECD loss rate at 1-month visit (*p* < 0.05) in both subgroups of the AFS group than in the GFS group, implying higher intraoperative comfort levels and less corneal damage of the AFS group with a low IOP setting.

**Conclusion:**

Owing to a lower IOP setting, Centurion^®^ Vision System with Active Sentry handpiece causes less corneal damage and pain perception during phacoemulsification for patients with low pre-operative ECD.

**Clinical Trial Registration:**

https://www.chictr.org.cn, identifier ChiCTR2300077865.

## Background

Cataract is the major cause of blindness worldwide and its operation rate is increasing. Cataract surgeries consist of phacoemulsification, extracapsular cataract extraction (ECCE) as well as intracapsular cataract extraction (ICCE). Among these options, phacoemulsification has become the mainstream because of its high efficiency and minimum harm to the cornea. It is reported that the loss rate of pre-operatively normal corneal endothelial cell density (ECD) caused by phacoemulsification is about 5∼18.7% (about 143.2∼543.4 cells/mm^2^) (1, 2). For most patients, such a small amount of ECD loss is negligible, but for patients with corneal endothelial diseases, it may cause irreversible corneal edema and even bullous keratopathy. As a result, to find a safer strategy during phacoemulsification for these patients has a profound clinical significance.

In the past, due to the limitations of fluidics control technology, it was necessary to significantly improve intraocular pressure (IOP) during cataract surgery to ensure the stability of the anterior chamber. However, the eyes would be exposed to pressure many times higher than the physiological IOP and go through certain complications such as corneal edema (3), retinal injury (4) and ocular pain (5). To solve these problems, Alcon Active Sentry^®^ technology integrates a pressure sensor into the phaco-handpiece to detect the surge happening. Once detected, the system opens the Quick Valve™ in the cassette to provide additional infusion fluidics to the phaco-tip, thus greatly stabilizing the anterior chamber (6) and enabling surgeons to complete the operation at a more physiological IOP level. This study intends to evaluate the safety of the active fluidics system (AFS) and the gravity fluidics system (GFS) under different IOP settings of Alcon Centurion^®^ platform as well as their damage to the cornea of patients with low pre-operative ECD.

## Materials and methods

### Subjects

This was a randomized controlled trial (RCT) and approved by the ethical committee of Peking University Third Hospital (M2022297). The study was conducted in accordance with the tenets of the declaration of Helsinki. Patients aged 60 to 85 years old with senile cataracts and a decrease in the corneal endothelial cell density (ECD) were selected and randomly divided into the AFS group and the GFS group, after which accepted phacoemulsification at Peking University Third Hospital from September 2022 to July 2023. The lens nuclear grade of the enrolled patients was grade 2 or 3 according to the lens opacities classification system III (LOCSIII). At the same time, their ECD was within 500∼1500 cells/mm^2^. Severe corneal edema, shallow anterior chamber depth (< 2.0 mm), short axial length (< 23 mm), diabetes mellitus as well as severe fundus diseases such as retinal detachment, macular edema, uveitis and silicon oil tamponade were excluded. The post-operative follow-up was 1 month.

### Surgical parameters

Forty eyes of the AFS group and 32 eyes of the GFS group were enrolled. Surgeries were all performed by a professional surgeon (X. Chen) with the Alcon Centurion^®^ platform (Alcon Surgical, Texas, USA) under either the active infusion mode with an Active Sentry™ handpiece and a Balanced tip (Alcon Surgical, Texas, USA) or the gravity infusion mode with an Ozil ™ handpiece and a Balanced tip. The energy settings were referred to Table 1.

**TABLE 1:** Preset Phacodynamic Parameters.

	Active fluid system (*n* = 40)	Gravity fluid system (*n* = 38)
IOP	30 mmHg	80 cm H_2_O
Torsional US Power ^a^ (%)	100	100
Vacuum (mmHg)	400	400
AFR (cc/min)	40	40

US, ultrasonic, ^a^ linear mode, AFR, aspiration flow rate.

The surgery was performed under topical anesthesia with 0.5% proparacaine. A corneoscleral incision was made on 12 o’clock of the cornea with a 2.2 mm keratome blade. The “soft shell” technique with two functional viscoelastic devices (Alcon DuoVisc^®^/VisCoat^®^, Texas, USA) were used to support the anterior chamber as well as protect the corneal endothelium. Continuous curvilinear capsulorhexis (5∼5.5 mm in diameter) was made and the phaco-tip was inserted to the anterior chamber through the corneoscleral incision to perform phacoemulsification. Hydrophobic intraocular lenses (Alcon AcrySof™ IQ, Texas, USA) were inserted into capsular bags. After the viscoelastic devices were replaced by perfusion fluidics, the corneoscleral incision was closed automatically without hydrating the anterior stroma.

A numerical rating scale (NRS) was used immediately at the end of the surgery to measure the intraoperative pain of the patient. Intraoperative parameters, including cumulative dissipated energy (CDE), total case time, estimated fluidics usage (EFU), ophthalmic viscoelastic devices (OVDs) usage as well as patient pain levels were recorded. Topical eye drops of 0.3% levofloxacin, 1.0% prednisolone acetate and 0.1% diclofenac sodium were used for 1 month post-operatively.

### Clinical examinations

Routine ophthalmological examinations including IOP, best corrected visual acuity (BCVA) and slit-lamp examination were performed before surgery and 1-day, 1-week, 1-month after surgery. Central corneal thickness (CCT) was calculated via an anterior segment optical coherence tomography (AS-OCT) (Casia2, Tomey, Japan) to evaluate corneal edema. Corneal edema is defined as an increase of more than 10% CCT, and can be further classified as mild (increase of 10∼20% CCT), moderate (increase of 20∼30% CCT) and severe (increase of more than 30% CCT) corneal edema (7). A confocal microscopy (Confoscan 4, Nidek, Japan) was used to observe corneal endothelial cell morphology and calculate ECD under the manual mode. Five discontinuous areas were selected with 4 peripheral corneal area and 1 central corneal area and the ECD was calculated as the mean of the five areas.

### Sample size calculation

Power analysis was used for sample size calculation. In the pilot study, P1D CCT increase of the AFS group was 4.9 ± 1.9%, while the data was 11.9 ± 4.8% of the GFS group. Difference in means was 7%. Assumed difference between the means is 4% which is about half of the difference in means in the pilot study. Bigger SD (4.8%) was selected from the GFS group as the assumed SD in sample size calculation. Other data: α(unilateral): 0.025, 1-β: 0.8, lost rate of follow-up: 10%.

### Statistical analysis

We used the SPSS 23.0 for Mac (IBM Corp., Armonk, NY, USA) for statistical analysis. The normal distribution and homogeneity of variance were first tested. Independent t tests were performed to compare energy parameters, age, ACD, CCT, ECD, and BCVA between the two groups. Chi-square tests were performed to compare the ratios between the two groups. And the difference was considered statistically significant when *p* < 0.05.

## Results

The demographic parameters of the 78 eyes (53 patients) were recorded in Table 2. The baseline parameters of the pre-operative data between the two groups were not significantly different (*p* > 0.05), so were the intraoperative parameters such as the total case time, CDE, EFU and the total OVDs usage (*p* > 0.05, Table 3). However, the patient pain level graded by NRS showed significant differences between groups, indicating higher pain levels of the GFS group (*p* < 0.0001).

**TABLE 2:** Demographic parameters.

	Active fluid system (*n* = 40)	Gravity fluid system (*n* = 38)	*p*-value
Age	71.34 ± 7.52	69.92 ± 6.89	0.375
Patient number	28	25	
Gender (Male/Female)	15(53.6%)/13(46.4%)	12(48.0%)/13(52%)	0.686
Eye (right/left)	16(40.0%)/24(60.0%)	18(47.4%)/20(52.6%)	0.512
**Stage of Nucleus** ^a^			
2	15(37.5%)	12(31.6%)	0.582
3	25(62.5%)	26(68.4%)	
ACD (mm)	3.03 ± 0.34	3.06 ± 0.52	0.545
CCT (μm)	541.1 ± 35.2	536.5 ± 31.2	0.419
ECD (/mm^2^)	1213.6 ± 223.5	1256.1 ± 191.3	0.667
Pre-operative BCVA, LogMAR	0.31 ± 0.25	0.30 ± 0.25	0.903
**History**			
FECD (stage I)	7 (17.5%)	5 (13.2%)	0.575
Glaucoma	15 (37.5%)	18 (47.4%)	
PPCD	2 (5.0%)	0 (0.0%)	
corneal endothelitis history	8 (20.0%)	9 (23.7%)	
no history	8 (20.0%)	6 (15.9)	

^a^ nucleus grading by LOCSIII. ACD, anterior chamber depth; CCT, corneal central thickness; ECD, endothelial cell density; FECD, Fuchs endothelial corneal dystrophy; PPCD, posterior polymorphous corneal dystrophy.

**TABLE 3:** Comparison of intraoperative parameters between groups.

	Total (*n* = 78)			Grade 2 nucleus (*n* = 27)			Grade 3 nucleus (*n* = 51)		
	**Active (*n* = 40)**	**Gravity (*n* = 38)**	***P*-value**	**Active (*n* = 15)**	**Gravity (*n* = 12)**	***P*-value**	**Active (*n* = 25)**	**Gravity (*n* = 26)**	***P*-value**
Total case time (seconds)	323.5 ± 151.7	372.3 ± 169.8	0.512	309.8 ± 153.1	345.5 ± 177.1	0.722	331.7 ± 156.2	384.7 ± 172.6	0.757
CDE (%-seconds)	5.52 ± 4.91	6.77 ± 5.52	0.613	1.93 ± 2.12	2.32 ± 2.57	0.587	7.67 ± 6.27	8.82 ± 7.91	0.498
EFU (ml)	38.0 ± 19.8	41.2 ± 28.5	0.719	29.9 ± 9.2	33.1 ± 12.8	0.439	42.9 ± 24.2	44.9 ± 35.7	0.951
OVDs usage (ml)	0.69 ± 0.54	0.72 ± 0.62	0.343	0.62 ± 0.33	0.66 ± 0.43	0.719	0.73 ± 0.71	0.75 ± 0.77	0.677
**Patient pain level**									
No pain	33(82.5%)	11(28.9%)	<0.0001	14(93.3%)	3(25.0%)	0.0001	19(76.0%)	8(30.8%)	0.005
Mild pain	6(15.0%)	19(50.0%)		1(6.7%)	6(50.0%)		5(20.0%)	13(50.0%)	
Moderate pain	1(2.5%)	8(21.1%)		0(0%)	3(25.0%)		1(4.0%)	5(19.2%)	
Severe pain	0(0%)	0(0%)		0(0%)	0(0%)		0(0%)	0(0%)	

CDE, cumulative dissipated energy; EFU, estimated fluid usage; OVDs, ophthalmic viscoelastic devices.

**FIGURE 1:**
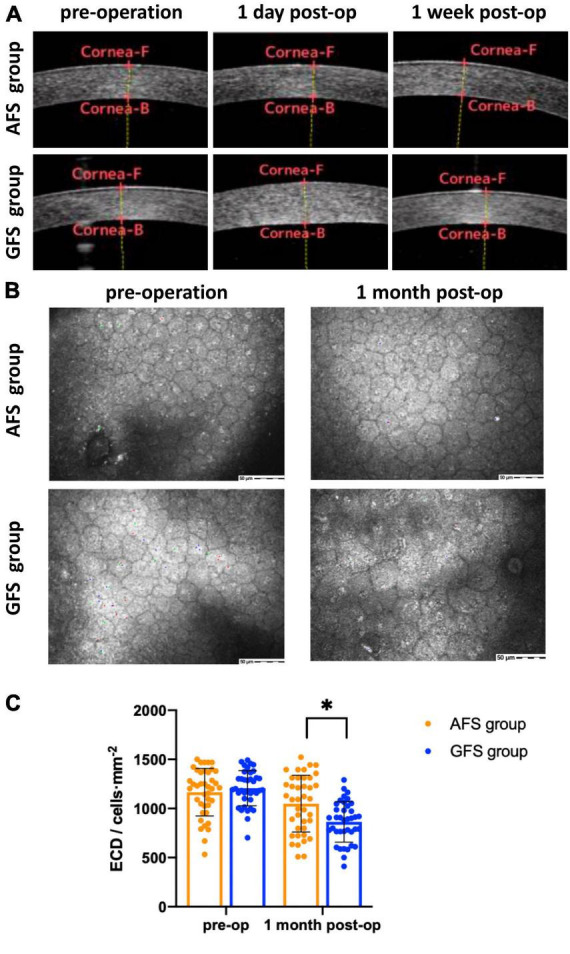
AS-OCT **(A)**, confocal microscopy **(B)** results and statistical analysis of ECD **(C)**. Corneal edema can be seen in GFS group at 1-day visit panel **(A)**. ECD decreased in GFS group at 1-month visit panel **(B)**. ECD of the AFS group was significantly higher than the GFS group at 1-month visit panel **(C)**. *p < 0.05.

Best corrected visual acuity (LogMAR) at 1-day visit was 0.10 ± 0.11 for the AFS group and 0.27 ± 0.18 for the GFS group, the difference was significant (*p* < 0.0001, Table 4). BCVA of the GFS group at 1-week visit improved to 0.11 ± 0.13, and the difference between groups was not significant (*p* > 0.05, Table 4). Also, BCVA at 1-month visit were not significantly different between groups (*p* > 0.05, Table 4). Stratified analysis was carried out according to the stage of the nucleus and showed that the above corneal edema rate at 1-day visit were statistically higher in the GFS group for both of the grade 2 and 3 nuclei subgroups (*p* < 0.05, Table 4). Change in CCT at 1-day visit and loss rate of ECD at 1-month visit of the GFS group were found to be significantly higher than the AFS group for both subgroups (*P* < 0.01, Figure 1, Table 4). No intraoperative or post-operative complications such as posterior capsule rupture, IOP elevation and endophthalmitis, etc., were observed within 1-month follow-up.

**TABLE 4:** Post-operative outcomes.

	Total (*n* = 78)			Grade 2 nuclei (*n* = 27)			Grade 3 nuclei (*n* = 51)		
	**Active (*n* = 40)**	**Gravity (*n* = 38)**	***P*-value**	**Active (*n* = 15)**	**Gravity (*n* = 12)**	***P*-value**	**Active (*n* = 25)**	**Gravity (*n* = 26)**	***P*-value**
P1D BCVA, LogMAR	0.10 ± 0.11	0.27 ± 0.18	<0.0001	0.10 ± 0.10	0.25 ± 0.16	<0.0001	0.10 ± 0.12	0.28 ± 0.19	<0.0001
P1W BCVA, LogMAR	0.08 ± 0.09	0.11 ± 0.13	0.127	0.08 ± 0.09	0.11 ± 0.14	0.135	0.08 ± 0.09	0.11 ± 0.13	0.155
P1M BCVA, LogMAR	0.08 ± 0.10	0.10 ± 0.13	0.428	0.08 ± 0.08	0.10 ± 0.11	0.399	0.08 ± 0.11	0.10 ± 0.16	0.532
**P1D corneal edema**									
None	31(77.5%)	11(28.9%)	<0.001	13(86.7%)	3(25.0%)	0.004	18(72.0%)	7(26.9%)	0.018
Mild	7(17.5%)	19(50.0%)		2(13.3%)	6(50.0%)		5(20.0%)	14(53.8%)	
Moderate	2(5.0%)	7(18.4%)		0(0.0%)	3(25.0%)		2(8.0%)	4(15.4%)	
Severe	0(0.0%)	1(2.6%)		0(0.0%)	0(0.0%)		0(0.0%)	1(3.8%)	
**P1W corneal edema**									
None	39(97.5%)	37(97.4%)	0.971	14(93.3%)	12(100.0%)	0.362	25(100.0%)	25(96.2%)	0.322
Mild	1(2.5%)	1(2.6%)		1(6.7%)	0(0.0%)		0(0.0%)	1(3.8%)	
Moderate	0(0.0%)	0(0.0%)		0(0.0%)	0(0.0%)		0(0.0%)	0(0.0%)	
Severe	0(0.0%)	0(0.0%)		0(0.0%)	0(0.0%)		0(0.0%)	0(0.0%)	
P1D CCT (μm)	579.2 ± 122.1	618.6 ± 157.9	0.015	569.8 ± 98.7	608.6 ± 113.3	0.039	584.8 ± 132.5	623.2 ± 192.2	0.011
Change of P1D CCT (%)	7.0 ± 3.1	15.3 ± 6.2	0.031	5.3 ± 2.2	13.4 ± 5.9	0.023	8.1 ± 3.9	16.2 ± 7.0	0.003
P1W CCT (μm)	555.9 ± 109.2	578.3 ± 131.1	0.159	549.1 ± 94.1	558.1 ± 99.8	0.447	560.0 ± 119.9	587.6 ± 146.8	0.274
Change of P1W CCT (%)	2.7 ± 2.5	7.8 ± 6.8	0.112	1.5 ± 1.2	4.0 ± 3.6	0.129	3.5 ± 2.2	9.4 ± 7.7	0.258
P1M ECD	1056.3 ± 294.2	863.7 ± 205.2	0.022	1089.8 ± 378.9	853.3 ± 291.5	0.032	1036.2 ± 209.2	868.5 ± 223.3	0.007
Loss rate of ECD (%)	13.0 ± 4.2	31.2 ± 9.8	0.018	12.0 ± 3.5	33.1 ± 8.9	0.009	13.6 ± 4.8	30.4 ± 10.3	0.003

CCT, corneal central thickness; ECD, endothelial cell density; P1D, one day after surgery; P1W, one week after surgery; P1M, one month after surgery.

## Discussion

The increase in CCT at 1-day visit and the loss rate of ECD at 1-month visit are short and medium-term corneal endothelial damage indicators, respectively, for cataract surgeries. In this study, we saw a significantly larger increase of (15.3 ± 9.2)% in CCT at 1-day visit for the GFS group while it was only (7.0 ± 3.1)% for the AFS group (*p* < 0.05). Similarly, the loss rate of ECD at 1-month visit for the GFS and AFS group was (31.2 ± 8.8)% and (13.0 ± 4.2)% respectively, and the difference was significant (*p* < 0.05). Moreover, when we further divide each group into grade 2 and 3 nuclei sub-groups, we found that the above difference existed in both sub-groups (*p* < 0.05). The above results showed that the AFS group with a low IOP setting made less corneal damage, regardless of the grading of the nucleus. From the traditional point of view, the corneal damage is mainly caused by the ultrasound energy, the beating of the nuclear fragments and the free radicals associated with ultrasound oscillation (8). However, in this study, the above parameters were comparable between the two groups considering the phaco-tips used, the nuclear grading as well as the intraoperative parameters of the two groups all had no significant differences. Then, it could be inferred that this difference in corneal damage was caused by different IOP settings during operation.

Intraoperative IOP is determined by a balance between the irrigating pressure and the outflow volume. Previous studies have found that the bottle height during phacoemulsification correlated with corneal endothelial cell loss (ECL) both *in vitro* and *in vivo*. Wenzel et al. performed intermittent irrigation and aspiration (I/A) in porcine eyes with varying bottle height from 100 to 150 cm and found that ECL was significantly increased from (−9.69 ± 4.81)% to (−21.99 ± 6.70)% by the bottle height elevation (9). In a clinical study, Suzuki et al. found that corneal ECL was significantly lower in the bottle height 30 cm group than in the 60 cm group at all time points until 3 months after surgery and concluded that phacoemulsification with a low bottle height was less harmful to the corneal endothelium (3). However, the above studies only focused on subjects with normal pre-operative ECD. In other words, the effects of intraoperative IOP to corneal endothelium with low pre-operative ECD remains to be elucidated.

Many diseases like iridocorneal endothelial (ICE) syndrome, Fuchs endothelial corneal dystrophy (FECD), posterior polymorphous corneal dystrophy (PPCD), glaucoma as well as corneal endothelitis may result in low corneal ECD. And these unhealthy endothelial cells in our study may have been undergoing greater stress during cataract surgery. Unlike other phaco-dynamic parameters that have been widely studied and optimized for corneal protection, intraoperative IOP setting has not yet been paid enough attention in cataract surgery. The most frequently used intraoperative IOP setting under traditional GFS is about 80∼100 cmH_2_O (approximately 59.2∼74 mmHg), in order to secure the anterior chamber’s stability. However, the physiological IOP is only 10∼21 mmHg, and a 60 mmHg or higher IOP may cause irreversible endothelium damage and even corneal edema. Núñez-Álvarez et al. induced mechanical damage to the corneal endothelium by simply elevating IOP and observed apoptosis of the endothelial cells (10). Corneal endothelial cells are sensitive to high IOP and pressure related damage, and this damage may be even greater to unhealthy endothelium with low ECD. Thus, relatively lower IOP settings or even physiological IOP settings during phacoemulsification may be vital to the corneal endothelium protection. The actual intraoperative IOP with AFS cannot be directly detected or calculated by formulas. But according to Nicoli et al. (11), their experimental research used an acrylic test chamber model to mimic the anterior chamber of the human eye and detected the actual intraoperative IOP using an electronic pressure transducer (Foxboro, Honeywell). They found that the actual intraoperative IOP with AFS maintained the same as target IOPs across a range of aspiration flow rates, indicating AFS’s ability to secure a more stable anterior chamber compared with GFS.

Except for the safety of the two fluidics systems, another focus of this study is the pain conception, or in other words, the comfort level of the patients during surgery. In cataract surgery, patients will have been undergoing varying degrees of discomfort even if local anesthesia has already been used. A study by O’Brien et al. showed that the average pain score observed during phacoemulsification was 1.18 ± 1.78 using the visual analog scale (12). In addition, Hou et al. published a case series of 136 patients, nearly half of whom experienced pain during the perfusion procedure and they found that reducing IOP by lowering the infusion bottle height, would relieve pain (5). In our study, 82.5% patients in the AFS group with low IOP settings experienced no pain during surgery, while the percentage was only 28.9% in the GFS group with normal IOP settings (*p* < 0.0001). Our results were in accordance with the above studies and emphasized again the importance of a low IOP setting to acquire higher comfort levels for patients during cataract surgery.

One shortfall of our study is that, because of the high long-term lost rate of follow-up, our follow-up period was relatively short and was only 1 month, thus making the observation of long-term post-operative results impossible. In conclusion, due to the reduction of dependence on high infusion pressure to stabilize the anterior chamber, the Alcon Centurion^®^ active fluidics system is able accept a lower IOP setting. And owing to that lower IOP setting, Centurion^®^ Vision System with Active Sentry handpiece causes less corneal damage and pain perception during phacoemulsification for patients with low pre-operative ECD.

## Data availability statement

The original contributions presented in this study are included in the article/supplementary material, further inquiries can be directed to the corresponding author.

## Ethics statement

The studies involving humans were approved by the ethical committee of Peking University Third Hospital. The studies were conducted in accordance with the local legislation and institutional requirements. The participants provided their written informed consent to participate in this study.

## Author contributions

YL: Data curation, Writing − original draft. JH: Conceptualization, Writing − review and editing. XC: Funding acquisition, Investigation, Writing − review and editing.
